# Assessing the Effectiveness of Tuberculosis Management in Brushtail Possums (*Trichosurus vulpecula*), through Indirect Surveillance of *Mycobacterium bovis* Infection Using Released Sentinel Pigs

**DOI:** 10.1155/2014/361634

**Published:** 2014-04-02

**Authors:** G. Nugent, I. J. Yockney, E. J. Whitford, M. L. Cross

**Affiliations:** Landcare Research, P.O. Box 40, Lincoln 7640, New Zealand

## Abstract

In New Zealand, wild pigs acquire *Mycobacterium bovis* infection by scavenging tuberculous carrion, primarily carcasses of the main disease maintenance host, the brushtail possum (*Trichosurus vulpecula*). We investigated the utility of captive-reared, purpose-released pigs as sentinels for tuberculosis (TB) following lethal possum control and subsequent population recovery. Within 2-3 years of possum control by intensive poisoning, TB prevalence and the incidence rate of *M. bovis* infection in released sentinel pigs were lower than in an adjacent area where possums had not been poisoned. Unexpectedly, TB did not decline to near zero levels among pigs in the poisoned area, a fact which reflected an unanticipated rapid increase in the apparent abundance of possums. Monitoring infection levels among resident wild pigs confirmed that TB prevalence, while reduced due to possum control, persisted in the poisoned area at >20% among pigs born 2-3 years after poisoning, while remaining >60% among resident wild pigs in the nonpoisoned area. When fitted with radio-tracking devices, purpose-released pigs provided precise spatial TB surveillance information and facilitated effective killing of wild pigs when employed as “Judas” animals to help locate residents. Sentinel pigs offer value for monitoring disease trends in New Zealand, as TB levels in possums decline nationally due to large-scale possum control.

## 1. Introduction

Bovine tuberculosis (TB) has been difficult to control in New Zealand livestock, primarily because the disease has become established in introduced mammalian wildlife [1]. The major wildlife maintenance host is the brushtail possum (*Trichosurus vulpecula*) [2, 3]. Although other wildlife species (including ferrets, deer, and wild pigs) can acquire* Mycobacterium bovis* infection, sometimes to a high prevalence level [4], they generally cannot maintain the infection cycle independently (i.e., they are spillover hosts which primarily acquire infection from possums [5]). Despite the impediment of a wildlife reservoir, there has been major progress toward TB freedom in New Zealand livestock and wildlife, as the result of a coordinated programme combining intensive lethal control (by poisoning and trapping) of possums over about 8 million ha (35% of the country) with livestock movement control and compulsory diagnostic testing (and slaughter of test positive animals [6]). Over the period 1995–2012, these combined approaches successfully reduced the number of infected cattle and deer herds by >95% [6].

As a result of that success, the aim of TB management in New Zealand changed in 2011 from a goal of achieving the Office International des Épizooties (OIE) international standard for declaring official TB freedom (a national-level period prevalence in livestock of <0.2% p.a. for 3 years) to one of progressive or “roll-back” eradication of TB from wildlife over 2.5 million hectares by 2026 [6, 7]. Under this goal of regional-scale eradication, areas will be declared free of TB in possums based partly on the outputs from a spatially explicit and quantitative “Proof of Freedom” (PoF) predictive modelling framework [8, 9]. This framework is in turn based, in large part, on empirical field surveillance data of TB levels in wildlife. However, direct surveillance of possums (through capture, necropsy, and mycobacterial culture) has become expensive, because possum densities are now generally very low and because this approach is often impractical in rugged terrain that is difficult to traverse on foot, prompting investigation of other ways of assessing the likelihood of TB persistence in possums. In this context, nonmaintenance spillover hosts can be used as sentinels for detecting* M. bovis* infection among wild animals in a given environment [5]. In New Zealand, wild pigs (*Sus scrofa*) have emerged as particularly sensitive sentinels for detecting persistent* M. bovis* infection in sympatric wildlife, since they are highly susceptible to becoming infected by scavenging tuberculous carrion (including possum carcasses [10, 11]). Pigs develop readily identifiable tuberculous lesions in primary predilection sites draining the oropharyngeal cavity, especially the submaxillary lymph nodes [12], but despite high levels of infection they seldom pass on* M. bovis* infection to conspecifics under New Zealand conditions [13], even though that does occur in some parts of Mediterranean Europe [14]. Carrion-feeders/scavengers have been used as sentinels for TB in other wildlife maintenance hosts, for example, coyotes (*Canis latrans*) in North America being used to indicate persistent* M. bovis* infection in white-tailed deer (*Odocoileus virginianus* [15, 16]). A recent report has suggested that Virginia opossums (*Didelphis virginiana*) may also provide surveillance potential for persistent TB in North America [17].

As sentinels, wild pigs have been shown to be far more sensitive indicators of the presence of TB in possums than ferrets or wild deer, the other two spillover species in New Zealand that could potentially be used as TB sentinels [5, 18, 19]. The high utility of pigs as a landscape-wide surveillance tool appears to reflect not only a remarkable ability to find possum carcasses [20], but also the very large areas that pigs cover; pig home ranges can be tens to hundreds of times larger than those of possums [21], providing extensive surveillance coverage. As a result, use of wild pigs as sentinels for TB surveillance has burgeoned in recent years, with approximately 13,000 pigs surveyed by TBFree New Zealand (the agency responsible for TB management in New Zealand) over the five years to mid-2012 [22]. This operational surveillance is based almost exclusively on collection and necropsy of resident wild pigs, killed by either recreational or contracted hunters. Although inexpensive, this approach produces only limited information about the duration and spatial extent of the TB surveillance each pig can provide: generally only the location point at death is known, so surveillance coverage is inferred retrospectively by fitting a circular average home range centred around that point. In reality such a fit will only rarely be an accurate depiction of the area each pig truly “surveyed” during its life. Sometimes, in the crucial cases where TB is actually detected in a wild sentinel, the age at death of that sentinel pig can be estimated to provide an indication of how recently* M. bovis* infection was acquired; however, this is not usual practice in surveillance operations since it incurs extra work effort and cost if conducted routinely (specifically, it requires the removal of the jaws, cleaning/sterilisation of the hard tissue, and laboratory-based assessment of dentition patterns). Moreover, for older pigs in particular, this form of age estimation provides only a broad indication of when infection was likely to have been acquired. In an effort to overcome these limitations, we have previously explored the use of deliberately released TB-free sentinel pigs [12]. In that study, translocated feral pigs were fitted with VHF radio transmitters and tracked at regular intervals for some months in a TB endemic area before being recovered, by which time all of them had become infected. This approach provided a more accurate and precise indication of where and when TB was acquired by the released sentinels than would have been obtained from wild sentinel pigs in cross-sectional surveying.

In the study reported here, we aimed to refine the released sentinel pig methodology for TB surveillance in three main ways. First, we used purpose-reared feral-type pigs raised in captivity, to avoid the difficulties of capturing and translocating suitable sentinels from distant locations where TB was absent. Secondly, we used GPS telemetry to provide a better depiction of the area covered by the pigs after release. Thirdly, we investigated whether the released sentinels could also be used to increase surveillance efficiency overall, that is, by using them to help hunters locate resident pigs, the so-called “Judas” technique that has been used previously to increase the efficiency of population control for pigs [11]. To evaluate the utility of these potential enhancements, we used captive-reared, purpose-released pigs as sentinels to detect TB in an area with endemic disease and compared trends in infection among these animals with those observed among wild pigs resident in the same area over the same time. The study was conducted in a remote part of the northern South Island high country of New Zealand where possum control was imposed (using a single aerial poisoning operation) for the first time in 2008. We focus primarily on assessing how the effectiveness of such possum control in reducing or eliminating TB could be most accurately monitored by assessing changes in disease status among pigs.

## 2. Methods

### 2.1. Study Area and TB History

**Figure 1:**
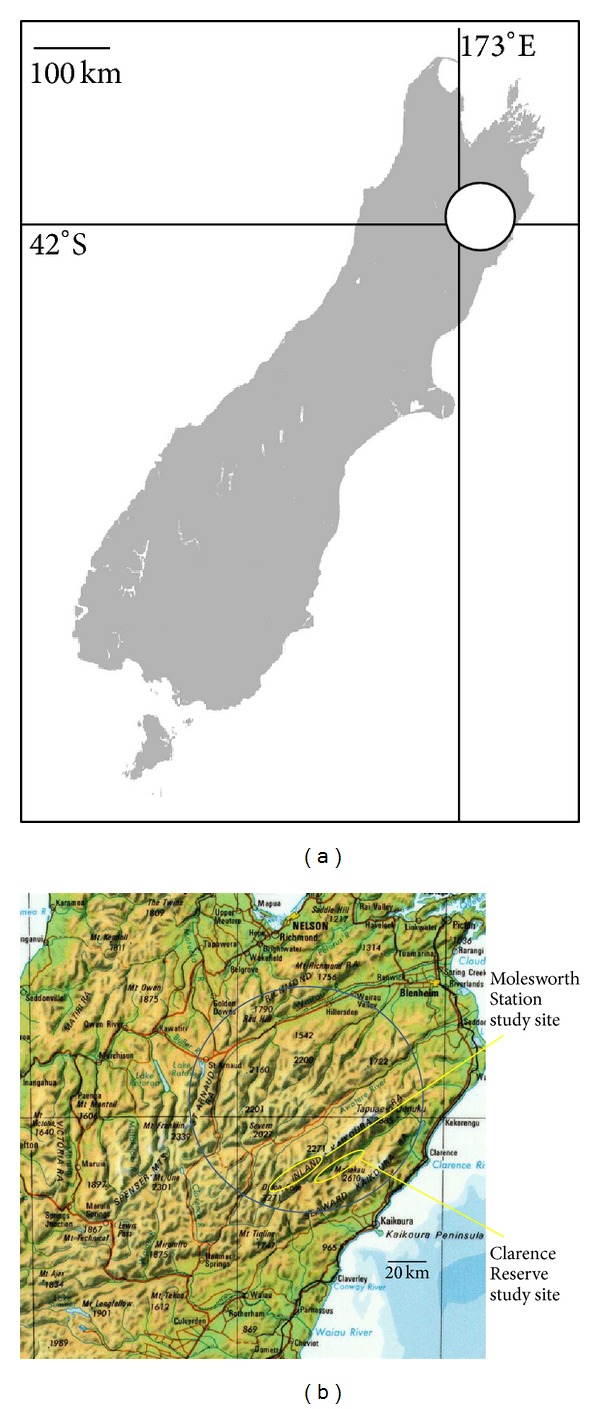
Outline map of the South Island of New Zealand highlighting the northern South Island high country region (circled), within which the Molesworth Station and Clarence Reserve study sites are located.

Between 2008 and 2012, we monitored changes in abundance in a possum population and assessed the effect of those changes on TB outcomes in free-ranging pigs (both purpose-released and resident). This was undertaken prior to and following the initiation of landscape-scale lethal control, in 2008, of a previously uncontrolled* M. bovis*-infected possum population in part of the 186,000 ha Molesworth Station (42.2°S, 173.3°E; Figure 1). The 2008 poisoning operation covered 28,500 ha of the southeast corner of Molesworth Station, immediately northwest of the Clarence River. The area is semiarid and largely unforested, comprising mountainous habitats (elevation range 550–2100 m) where the vegetation is dominated by low-lying shrubs and grasses, interspersed with rose briar, matagouri, and other scrubby plants (see Glen et al. [23] for full description). Such dryland regions cover 19% of New Zealand and are generally poor habitat, with possums inhabiting larger than usual home ranges [21, 23] and the regions supporting lower than usual population densities. A recent study recorded 44–69 possums/km^2^ in such dryland habitat, 5- to 29-fold lower than the densities expected in more suitable podocarp, broadleaved forest habitat [24]. The Molesworth Station study site and adjacent areas also contain a low to moderate density of feral pigs that is controlled to varying degrees by recreational hunting (1-2 pigs/km^2^ [21]).

Immediately southeast and adjacent to the Molesworth study area, the 37,000 ha Clarence Reserve (42.3°S, 173.4°E) was used as a comparative nontreatment area where no control was applied to possums. Separating the two sites is the Clarence River (average annual flow > 10 cumecs, a significant geographical barrier for possum movement between sites).

Tuberculosis has been recorded in possums and pigs over several decades in the Molesworth Station and Clarence Reserve areas, with particularly high levels of* M. bovis* infection recorded in pigs. A 2005 cross-sectional survey of TB prevalence in the unmanaged possum population in the study area and its surrounds recorded a 1.5% prevalence (95% CI 0.3–4.3; *n* = 203) of culture-confirmed* M. bovis* infection in possums [25]. In contrast, a series of cross-sectional surveys conducted between 1999 and 2007 indicated an average 69% prevalence (95% CI 48–91; *n* = 220) of TB in wild pigs [13, 26].

### 2.2. Possum Control Operation and Possum Monitoring

The possum population on Molesworth Station as a whole had been progressively brought under TB management during the 2000s, with the southeastern section study site being the last of the TB-affected parts of the station to be brought under possum control. An aerial poisoning operation was conducted there in October 2008, with the expectation that intensive culling would reduce possum numbers to below the density threshold for TB persistence for at least five years [27], after which further control would be imposed (as per current best practice for eliminating TB from possums in large remote areas) [6, 28]. The aerial poisoning operation used helicopters to distribute cereal baits containing 1.5 mg sodium monofluoroacetate (1080)/g at 1.0–1.5 kg of bait/ha [29–32] over the most possum-prone parts of the area. This selective coverage approach has traditionally been used in mountainous dryland areas, because possum densities at the highest altitudes (which are predominantly bare rock, scree, and shingle) are very low, if not zero. For the 2008 operation selective coverage was implemented more objectively, by targeting the areas where possum densities before control were thought to be above the likely threshold density for TB persistence. Briefly, selective coverage was based on a spatial map of predicted possum density at carrying capacity [26, 33, 34] as expressed by the relative abundance measure CKRI (capture and kill-rate index, the derivation of which is described in detail elsewhere [34]). The study area on Molesworth Station was divided into four blocks of 6000–9000 ha area each. In two of those, 1080 baiting was deployed to all habitat predicted to have a possum CKRI > 5% (which broadly approximates to the traditional practice of omitting high-elevation areas from possum control); in the other two reduced coverage blocks, baiting was deployed only to habitat predicted to have a CKRI > 10% (i.e., control was restricted to habitat predicted to hold the highest numbers of possums). Consequently, across the four study blocks, poison bait was deployed aerially to a total of 17,800 ha, about two-thirds of the study area.

### 2.3. Trends in Possum Relative Abundance Indices after Control

To assess the efficacy of the control operation in reducing possum density, we used two before-and-after measures of possum activity (i.e., the rate at which possums interfered with noninvasive detection devices) as a proxy for possum relative abundance (National Pest Control Agencies (NPCA) [35, 36]), as well as a single direct measure of possum relative abundance postcontrol [37]. This low cost approach was utilised because the rugged terrain of the study made fieldwork difficult and expensive. To measure possum activity, we used two interference detection devices, palatable ChewCards (CC) containing a food attractant [38] and unpalatable WaxTags (WT) which possums bite out of curiosity [39]. In each of the four blocks, at least 8 transects were established, with 20 pairs of devices (one CC and one WT placed ~10 m apart) spaced about 50 m apart along each. The percentage of each device type bitten was calculated for each transect and the resultant indices (CCIs and WTIs for ChewCards and WaxTags, resp.) were Poisson-transformed for analysis (as described previously [32]). For logistical and cost reasons, the period of device exposure varied between successive surveys but was always the same between blocks within surveys. The CCIs and WTIs were measured immediately before control in October 2008, immediately afterwards (November 2008) and then one (2009) and two years (2010) later.

In addition, for operational performance monitoring purposes, trap-catch rate monitoring was conducted in each block one month after control, to provide a direct measure of possum relative abundance. Leg-hold trapping was conducted following a trap-placement protocol that has been nationally standardised to provide consistent cross-study estimates of relative possum numbers after poison control [37]. The resulting residual trap-catch index (residual TCI) is a standardised index for gauging operational efficacy, with TCI values broadly representing possum true abundance values [40].

### 2.4. Monitoring “Judas” Sentinel and Resident Wild Pigs

To determine the effect of possum population control on TB levels in wild pigs and to compare the utility of released captive-reared sentinel pigs and resident wild pigs as TB surveillance tools, we conducted pig necropsy surveys in the poisoned study area on Molesworth Station and in the adjacent unpoisoned Clarence Reserve. The TB status of each pig was determined, along with duration of exposure (for released pigs) or age (for resident pigs). These data were used to calculate and compare the prevalence and incidence of infection between areas, between years, and between resident and released sentinels.

The purpose-reared released sentinel pigs were obtained from TB-tested breeding stock captured from the wild, either locally or from the Marlborough Sounds (designated a TB-free area) and raised on-site in enclosed pens near Molesworth Station. Eighty-two pigs were bred for release (mean age 8.1 months at release) and classified as free from prior mycobacterial infection after intradermal tuberculin testing (ear skin site) just prior to release. All animals had been fitted with surgically implanted VHF transmitters and/or “store-on-board” GPS collars for tracking purposes (see Yockney et al. [21] for methodology) and were liberated into the Molesworth and Clarence Reserve study sites in a series of releases between May 2009 and July 2010. Data downloaded from the recovered GPS devices were used to map the areas used by the sentinels during their time in the wild and to estimate home range size using a 95% isopleth fixed-kernel density estimator (KDE). Analyses were conducted in Home Range Tools for ArcGIS using the reference method of Seaman et al. [41], analysing a 95% kernel home range using a raster cell size of 12 m [42, 43]. All animal manipulations were covered by Landcare Research Animal Ethics Approval 08/07/03.

Samples of resident wild pigs were obtained either by collecting heads from pigs killed by recreational hunters between September 2009 and May 2012 or killed by contracted aerial shooting (from a helicopter) on hunting forays conducted over the same period. The aerial hunting was conducted using two approaches.

*(1) Unassisted Hunting.* This was conducted at the times of the year when pigs were most visible (predominantly late autumn and winter) but relied solely on the skill of the hunters to locate pigs. 

*(2) “Judas” Assisted Hunting.* For this, released sentinels pigs were periodically relocated aerially using VHF telemetry and any resident pigs associated with them were shot. This approach was used both before any sentinel was due to be recovered (in which case the sentinel was not shot) and also when the sentinel was being recovered (at which time the sentinels were also shot). In-depth explanation of this technique is provided elsewhere [11].

The numbers of pigs successfully located, killed, and necropsied were recorded for each method, along with the amount of helicopter flying time per foray as a proxy for hunting effort, and the kill rates were calculated and compared.

### 2.5. Pig Necropsy Procedures and TB Evaluation

Whole body necropsies for TB detection were conducted on some pig carcasses, mostly at one of several field processing sites in the study area. For most pigs, however, only the head was inspected, either at one of the field processing sites or in a purpose-designed necropsy facility at Landcare Research (Lincoln, New Zealand). Previous research has shown little or no loss of diagnostic sensitivity from restricting inspection to the head, because the head region lymphatic system is the predominant site of infection in pigs [13], and there is no reason to expect any minor loss of sensitivity that would invalidate comparisons between treatments.

Necropsies were conducted by experienced staff, using sterile techniques. For all pigs, inspection of the head involved removal and thin slicing (1–3 mm) of the submaxillary, parotid, retropharyngeal, and atlantal lymph nodes and the oropharyngeal tonsils. Tissue samples (up to a maximum of 3) of any gross lesions suggestive of TB were submitted for mycobacterial culture to AgResearch Ltd (Wallaceville, Upper Hutt, New Zealand), along with a tissue sample of the submaxillary lymph nodes from all pigs. Infected/noninfected status was assigned on the basis of culture-confirmed presence of viable* M. bovis* bacilli. Disease severity among animals was scored on a 0–7 point scale, as described previously [25] and outlined in the Supplementary Material (see Supplementary Material available online at http://dx.doi.org/10.1155/2014/361634); a score of 0 represented NVL (no visible lesion) cases with culture-negative results, 1 represented NVL cases with at least one culture-positive tissue, and ≥2 represented culture-positive lesioned animals, with progressive scores from 2 to 7 representing the greatest single dimension of the largest head-region lymph node gross lesion. For resident wild pigs, the jaw was also removed and, using the methods described by Clarke et al. [44], an age (in months) was assigned based on mandibular dentition, up to a maximum of 42 months (when full dentition is attained). An approximate birth date was estimated by subtracting the age from the kill date, and these data were used to assign pigs to latest likely birth-year cohorts, primarily to assess whether they were likely to have been born before or after possum control in late 2008.

The prevalence of infection was calculated for the various birth cohorts for each study area. However, for older resident pigs in particular, the presence of infection detected in pigs shot after possum control could have resulted from acquisition of infection before control. We therefore also calculated apparent annual incidence rates (AAI: estimated number of new infections per year of pig exposure [45]) to more accurately quantify and compare the rates at which pigs became infected in relation to when they were killed. For resident wild pigs, we assumed that piglets did not become fully susceptible to environmental* M. bovis* infection (through scavenging) until 2 months of age (the earliest likely weaning age for wild piglets [46]), and for each pig the exposure time (in months) was subsequently calculated as the estimated age minus 2 months, and as half that for infected pigs (assuming that infection had been acquired at a midpoint between age 2 months and necropsy). The AAI was then calculated for each sample group as the number of infections per pig year of total exposure [12]. For purpose-reared and released sentinels, the exposure period was simply the time between release and recovery (assuming full susceptibility to* M. bovis* infection from release date).

## 3. Results

### 3.1. Changes in Possum Abundance Indices after Control

**Figure 2:**
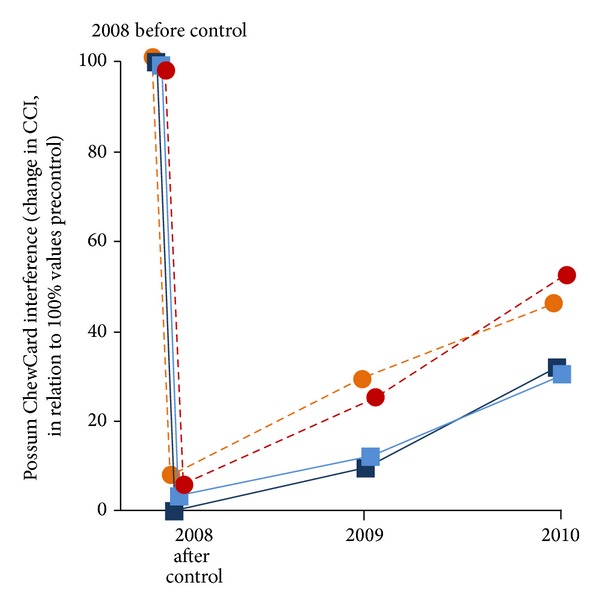
Changes in possum relative abundance indices, as determined by measuring changing levels of ChewCard interference, in four blocks on Molesworth Station, following aerial poisoning of possums in October 2008. Red lines refer to reduced coverage blocks (*n* = 2); blue lines refer to normal coverage blocks (*n* = 2).

Before poisoning in October 2008, CC and WT indices of relative abundance confirmed the presence of moderate numbers of possums in the study area (Table 1). Immediately after control, CCIs and WTIs were very low, as was the residual TCI, indicating that substantial reductions in possum density had been achieved (Table 1). Expressed relative to the precontrol indices (Figure 2), the efficacy of possum control was greater than 90% in all 4 blocks, although it appeared to be less effective overall in the reduced coverage blocks.

In the ensuing two years after possum control, the CCIs of possum interference with detection devices increased rapidly in all four blocks (Figure 2). One year after poisoning, the level of interference had increased (depending on block) to 11–27% of the precontrol indices, and within two years to 31–49% of the precontrol levels. Although the indices recorded two years after control were higher in the two reduced coverage blocks, this difference largely reflected the greater level of ChewCard interference recorded in those blocks immediately after control in 2008, as the postcontrol rate of increase in CCI was similar in all blocks regardless of poison coverage.

### 3.2. Necropsy Results for Pigs

Between 2009 and 2012, necropsy data were obtained from 421 pigs, comprising 51 purpose-reared sentinels (from a total of 82 radio-telemetered pigs originally released) and 370 resident wild pigs (of which 352 were assigned ages) (Table 2). Across all 421 pigs, just over half (215 cases) were culture positive for* M. bovis*; of these, 11 (5%) had no visible gross lesions, indicating a low incidence of subclinical infection among animals surveyed in this study.

### 3.3. Effectiveness and Utility of Released Sentinel Pigs

*Tuberculosis Monitoring.* The moderate level of recovery (51/82) of purpose-reared released sentinel pigs reflected a combination of telemetry equipment failure (resulting in pigs not being able to be relocated) or mortality of releases, either as a result of harsh winter conditions or as a result of being killed by recreational hunters. The 51 necropsied radio-telemetered sentinels released between May 2009 and July 2010 were subsequently recovered, on average, 8.1 months later. Released sentinels on Molesworth Station became infected in all four blocks, with AAIs varying between 23 and 42%; overall, 14 (27.5%) of released sentinel pigs became* M. bovis* infected (Table 3).

The TB prevalence and AAI for sentinels released into the poisoned areas on Molesworth Station were lower, after possum-control, than for those released into the Clarence Reserve (Table 3). To compare infection rates between released sentinels and resident wild pigs, a subset of 76 resident wild pigs that would have been newly weaned (i.e., reached 2 months of age) during the same May 2009 to July 2010 period was selected from the main wild pig data set (Table 3). Depending on area, culture-confirmed TB prevalence was 23–41% lower in released sentinels than in resident wild pigs, while AAI values were 18–33% lower (Table 3). Lesion severity scores were similar regardless of type of pig or study site.

*Surveillance-Related Movements.* Of the 30 purpose-reared pigs fitted with GPS devices and released, 14 failed to produce enough useful data, for a variety of reasons. The most common reasons were that the collar slipped off the pig at an early stage, that the GPS implant failed, or that the collar ran out of battery power well before its predicted lifespan. Two collars were never recovered, due to lack of a trackable signal, most probably due to the aerials being chewed by other pigs, which is a common occurrence.

**Figure 3:**
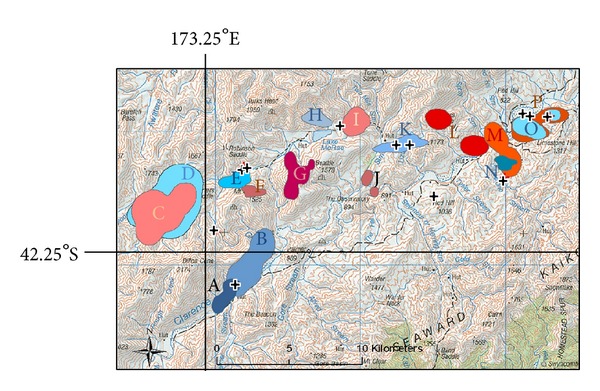
Fine detailed topographic map depicting location, shape, and size of the 95% kernel home ranges calculated from GPS units recovered from 16 purpose-reared sentinel pigs, released onto Molesworth Station and Clarence Reserve. Each individual pig's home range is depicted by a different colour and letter (*n* = 16). In relation to the areas covered by the GPS-tracked released sentinels, overlaid crosses represent kill locations for 12 confirmed TB-positive pigs (both released sentinels and resident wild pigs) shot during hunting forays.

Sixteen pigs (8 males, 8 females) provided sufficient data for home range analyses, comprising a total of 1412 days of GPS coverage. The average 95% KDE home range estimate for GPS-tracked pigs was 506 ha (range 103–1948 ha), with the largest home ranges recorded from pigs released into an area of largely open habitat where very few resident pigs reside. This average home range equates to a predicted circular area with a radius of 1.3 km; however, observed ranges were predominantly not circular (Figure 3). Following the process used to assess the spatial coverage normally provided by wild sentinel pigs when only their location at death is known [12] and overlaying a circle representing the assumed (= average, circular) home range size around the location at which a GPS-tracked released sentinel had been killed, the discrepancies between the actual and inferred coverage were usually large. The greatest discrepancies arose when a sentinel with a long but narrow actual range was killed at one end of that range (e.g., Pig A, Figure 3). Location fixes for the GPS-tracked sentinel pigs indicated that most pigs remained close to where they were released; average distance between release and kill site was 1.73 km (95% CI 1.1–2.3; *n* = 16), although one pig moved >6 km from its release point. Including non-GPS pigs, all but one of the released pigs remained within the block in which it was released.

**Figure 4:**
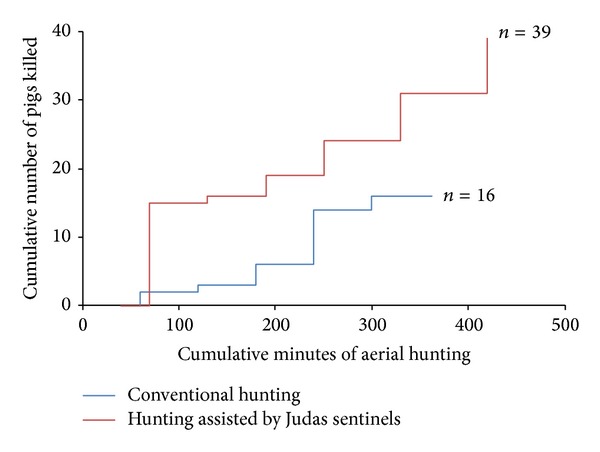
Comparison of killing rates for pigs, from six aerial hunting sessions on Molesworth Station when hunting conventionally or using radio-collared released “Judas” pigs to assist location of mobs. Assisted aerial hunting accounted for 39 pigs (31 resident wild animals and 8 purpose-released “Judas” animals; red line); unassisted hunting accounted for 16 wild pigs (blue line).

*“Judas” Utility.* Use of purpose-reared released pigs as “Judas” animals, to locate and kill the resident pigs with which they had associated, resulted in a substantial increase in aerial hunting efficiency compared with unassisted hunting (Figure 4). It required 7 hours of aerial hunting to kill 8 “Judas” pigs and 31 resident wild pigs, compared with 5 hours to kill 16 resident wild pigs during unassisted hunting. During the hunting phase, kill rates varied from 1 to 15 pigs/hour. The overall kill rate using “Judas” assisted hunting was 5.4 ± 1.7 kills/hour compared to 3.2 ± 1.24 kills/hour for unassisted hunting, which effectively halved the cost of obtaining resident pigs for surveillance purposes. Although not quantifiable, hunters considered that “Judas” assisted hunting was particularly useful in obtaining data from resident pigs in areas where there was extensive scrub cover (that would usually shield such residents from being shot there).

### 3.4. Changes in TB Levels among Resident Wild Pigs following Possum Control

Of the 352 resident wild pigs assigned to an age class, 201 (57%) were culture positive for* M. bovis*. The resident pigs shot between 2009 and 2012 were assigned to six birth-year cohorts, with the “2007 or prior” cohort group containing mostly fully adult pigs, while the 2012 cohort contained only recently born animals (reflected in the downward trend in the median age of successive cohorts) (Table 2). The 2012 cohort was excluded from subsequent analyses because of the small sample size and its very low median age.

**Figure 5:**
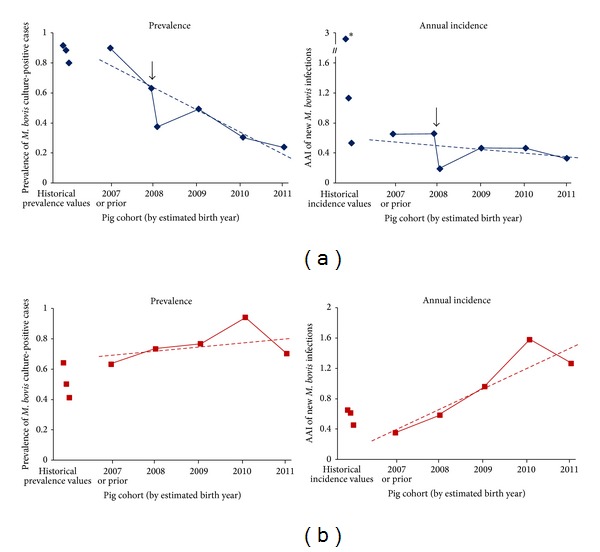
Trends in prevalence of culture-confirmed tuberculosis and of apparent annual incidence of new* M. bovis* infections among successive cohorts of pigs from Molesworth Station (a) and Clarence Reserve (b) study areas. Dashed lines represent 5-year linear trend lines fitted to the data from 2007 to 2011. Arrows refer to point of poison control of possums (Molesworth Station only) for which the 2008 cohort data were split into prepoison (Jan–Sept) and postpoison (Oct–Dec) portions. Historical prevalence/incidence values are presented for three sets of similar surveys conducted over the periods 1999–2003, 2003-2004, and 2005–2007; data in these historical surveys are presented for total pigs and are not differentiated according to birth year cohort. * includes one set of Molesworth surveys in 2005–2007 where predominantly yearling pigs were sampled (mean age 7.8 months, *n* = 40); hence an extremely high AAL value of 2.88 was recorded.

The prevalence of TB in resident wild pigs declined across the successive cohorts killed on Molesworth Station, from 90% in those born in or before 2007 to 24% in the 2011 cohort (Figure 5(a)). In contrast, TB prevalence in resident pigs from the unpoisoned Clarence Reserve was equal to or higher than in all later-born cohorts compared to the 2007 or prior cohort group (Figure 5(b)). The same divergence over time in the TB levels in resident pigs on Molesworth Station from those from the Clarence Reserve is evident in the trends in incidence, with AAI increasing across successive cohorts in the latter area but declining in the former (Figure 5). Although both the prevalence and AAI are biased (in opposite directions) by the differences in mean age between cohorts (Table 2), it is evident for the purposes of this paper that infection rates in pigs fell in the area that was poisoned relative to those in the area that was not poisoned. It is equally evident that infection rates in the poisoned study area did not fall to near zero levels, with new* M. bovis* infection cases still being recorded in Molesworth Station pigs born 2-3 years after possum control, albeit at reduced (but still substantial) annual rates of 0.47 or 0.33 new infections per pig for pigs born in 2010 and 2011, respectively (Figure 5(a)).

## 4. Discussion

Although this paper focuses primarily on assessing the effectiveness of purpose-reared/released sentinel pigs for TB monitoring, the possum-control management outcomes we document are important in interpreting the findings in pigs and further are themselves operationally important. In operational terms, the October 2008 culling was successful because it resulted in possum residual TCIs of <2%, which is the standard management target for such operations [37, 47]. Wildlife TB managers consider possum control operations that achieve this target do not need to be repeated for at least 5 years, because with the intrinsic (or maximum) exponential rate of annual increase of possums being of the order of 0.22–0.59 [48–50], the residual possum population is unlikely to recover within five years to levels where intraspecific* M. bovis* infection could once again be maintained by density-dependent transmission [27]. However, our indices of possum relative abundance here suggested apparent population recovery more quickly than the expected rates; the annual exponential increase in CCIs between 2008 and 2009 ranged between 1.28 and 1.68 per block (average 1.41) for the whole poisoned area, and between 2009 and 2010 ranged between 0.62 and 1.00 per block (average 0.85). These values appear to be beyond the reproductive capacity of the residual possum population. The explanation for this rapid increase in the indices is not known but could include some combination of (i) very high predominance of adult females amongst survivors, (ii) mass immigration of possums from the unpoisoned areas within each block, or (iii) an increase in the detectability of possums as a result of ranging more widely after poisoning in response to reduced densities. Although this is the first record of the phenomenon for an unforested area, it has been recorded previously in forested areas of New Zealand [50], including in areas far from the control areas' boundaries in which the entire area had been poisoned, making the second explanation above (mass immigration) unlikely.

Whatever the cause, it is clear that surviving possums were widespread in all four poison-treated blocks on Molesworth Station, with relative abundance indices higher than expected for the first two years after an apparently successful control operation. In relation to this, the moderate incidence of new* M. bovis* infections among resident wild pigs born up to three years after the poison operation and (more compellingly) among purpose-reared released pigs known to have spent their entire release period within the poisoned areas, provides an indication that TB was still present in the environment in the months and years immediately following poisoning. Although we did not directly monitor TB in possums themselves, we infer that, because the poison operation had not resulted in the sustained reduction in possum population density that was anticipated, a consequence of this was persistence of TB in that residual possum population, such that the expected rapid decline to near zero levels of TB in sympatric pigs did not occur here, as it has been observed to do elsewhere in New Zealand following intensive possum control [29]. However, by quickly identifying that the TB outcomes were not as good as expected, this study further demonstrates the utility of feral pigs as indicators of TB presence and that deliberately-released sentinels were of further use.

An innovation in this study was the on-site rearing of purpose-reared sentinel pigs for TB surveillance, which we showed to be feasible. Although more costly than capturing and translocating pigs from distant TB-free areas (see below), the approach enables far greater control over variables such as timing of releases and size of the pig at release; the latter is important where radio collars (rather than ear tags or implants) are used because it is difficult to fit collars to small (young) pigs that allow for subsequent growth yet are likely to remain attached. Some of the most obvious advantages of using released sentinels relate more to assessing the immediate effects of possum control on TB levels in wildlife (as in this study) than to PoF surveillance, at least where one or more of them become infected. Where that occurs, they provide a more accurate and precise delineation of where (and when) in the landscape TB is still likely to be present in possums than if sympatric wild pigs had been used. Firstly, in this study, the knowledge (from VHF and GPS telemetry) that released sentinels mostly remained close to where they were released enabled us to confidently infer that TB had persisted in all four Molesworth Station blocks, since the average distance between release and recovery of these sentinels was <2 km. Secondly, at a finer geographic scale, the identification here of accurate home range shapes for tuberculous purpose-reared and GPS-tracked sentinels allowed a more precise spatial identification of the likely infection source, compared to resident wild pigs, where only the location at death is known and a mean circular home range is applied centred on the kill location. Because pigs usually range according to geographical features (e.g., along river valleys) their actual area coverage is often more elongated than circular [51, 52] so, as was the case with Pig A in this study, if a pig is killed at one end of such a range, the presumed original infection source (a tuberculous possum carcass) may have been several km from where an assumed circular range might suggest. Hence, the GPS-telemetered pigs in particular were of greatest use in pinpointing likely foci of TB persistence in possums. This attribute will be particularly valuable when using pigs as surveillance tools in the future, as TB in possums becomes more sparse during eradication procedures and the need for rapid follow-up control of residual wildlife disease foci increases [8].

Offsetting those advantages, pigs raised in captivity and released as bespoke sentinels appeared to be somewhat less sensitive than pigs born in the wild, with the released sentinels acquiring* M. bovis* infection at lower rates than case-matched residents. The reasons for this are not known but could include (i) reduced ranging behaviour after handling and radio tagging for the purpose-reared sentinels, as has been reported in some other medium- and large-size wild mammals [53, 54]; (ii) a lack of familiarity with possum carrion as food; (iii) less help from other pigs in finding possum carrion (the captive-raised pigs were released in pairs, whereas when case-matched to predominantly yearling resident wild pigs for analysis, it is possible that the latter were still part of a cooperatively scavenging family group) [55]. Despite this apparent lower sensitivity, the purpose-reared released pigs provided crucial insight not readily available from residents, specifically, a guarantee (through radio-tracking) that they had remained within the study area, eliminating the possibility (albeit unlikely given the size of the area, the numbers infected, and the young age of some of the infected pigs) that some of the resident wild pigs shot in the poisoned area had acquired* M. bovis* infection elsewhere and then moved in to the poisoned areas.

In general, the use of wildlife sentinel species to monitor environmental disease trends has been reported elsewhere for a variety of zoonotic diseases [56–58] and for diseases of agricultural importance, including for monitoring bovine TB [15, 16]. However, such studies have focussed mainly on using wildlife sentinels to monitor patterns of geographic spread of infection within an environment and into previously unaffected areas. In contrast, in New Zealand, pigs are now mainly used (as in this study) to assess the effects of possum control on declining TB levels in wildlife [29] and, much more commonly, as a surveillance tool for indirectly assuring the absence of TB (as a result of previous possum control) from large areas [8, 22]. Almost all of this latter PoF surveillance is based on monitoring TB in fully wild pigs. Wild pigs are usually obtained from recreational or contracted hunters at a cost of $NZ50–300 per animal, including necropsy and culture of TB-like lesions (TBFreeNZ, unpublished data). In contrast, purpose-reared sentinel pigs cost approximately $NZ1500–2000 apiece to breed, raise, and radio-track, but they provide far more surveillance information. In addition, this study has shown that purpose-reared pigs also offer utility in terms of operational improvements during kill-out, which is a necessary end-point once the disease surveillance purpose of pigs has been expended. In this study purpose-reared and released “Judas” pigs were particularly useful in enabling a kill rate of resident wild pigs twice that achieved by unassisted aerial hunting—as flying costs are by far the greatest component of the cost for obtaining resident wild pigs using aerial hunting; this improved hunting efficacy effectively halved the associated costs. Although the “Judas” pig approach has been applied previously to increase the efficiency of pig population control through eradication [59] and for TB surveillance purposes [60], the present report is the first to use purpose-bred pigs for this purpose.

Overall, we conclude that purpose-reared, released and telemetry-tracked pigs provide useful TB surveillance and can also act effectively in the “Judas” context. They are likely to be most useful as an adjunct to surveys of resident wild pigs in operational contexts where the resident pigs are only patchily distributed across the area of interest and/or where there are large areas of dense cover that make it difficult to obtain sufficient numbers of resident pigs, for TB surveillance purposes, without “Judas” assistance. Whatever the explanation for the unexpectedly high persistence of TB on Molesworth Station, after what initially appeared to have been an effective possum control operation, our results suggest that control targets couched in terms of possum density indices may sometimes need to be set particularly low for dryland areas with sparse populations of widely ranging possums, in order to be able to break the TB cycle in possums quickly.

## Supplementary Material

In pigs, TB lesions were classified on a scale of 2–7 based on ascending lesion severity of the largest recorded lesion in the head region lymphatic system. In the absence of gross lesions, a score of 1 represented an animal bearing *M. bovis*-infected tissues while a score of zero represented a non-lesioned/non-infected animal.

## Figures and Tables

**Table 1:** Predicted (CKRI) and actual (residual TCI, CCI, and WTI) measures of the relative abundance of possums before and/or after aerial 1080 poisoning in four study blocks on Molesworth Station, 2008.

	Normal coverage blocks (*n* = 2)		Reduced coverage blocks (*n* = 2)	
	Precontrol	Postcontrol	Precontrol	Postcontrol
Predicted CKRI	Both > 5%	N/A	Both > 10%	N/A
Residual TCI	—	0.8%, 0.4%	—	1.8%, 1.1%
ChewCard index (CCI)	163%, 68%	3%, 0%	224%, 190%	18%, 11%
WaxTag index (WCI)	75%, 22%	0%, 0%	87%, 79%	7%, 6%

Each index represents a measure of possum relative abundance. CKRI: capture/kill-rate index (proportion of possums trapped and/or killed by cyanide paste relative to trap/poison frequency, as described previously [34]); TCI: trap-catch index (proportion of possums trapped relative to a standardised trap frequency and spacing pattern, as described previously [35]); CCI and WTI: ChewCard or WaxTag indices (possum interference/detection devices to detect possum presence and activity, as described previously [36–38]).

**Table 2:** Population age structure, gender, and TB lesion severity among resident wild pigs captured from Molesworth Station and Clarence Reserve between 2009 and 2012.

	Molesworth Station							Clarence Reserve						
Birth-year cohorts	2007 or prior	2008	2009	2010	2011	2012	Overall	2007 or prior	2008	2009	2010	2011	2012	Overall
Number (males, females)	30 (13m, 17f)	26 (12m, 14f)	66 (34m, 32f)	72 (34m, 38f)	21 (8m, 13f)	4^∗1^ (3m, 2f)	**T** **o** **t** **a** **l** ^∗2^ ** 221 pigs**	13 (9m, 4f)	25 (14m, 11f)	40 (20m, 20f)	16 (7m, 9f)	20 (6m, 14f)	2^∗1^ (1m, 1f)	**T** **o** **t** **a** **l** ^∗2^ **131 pigs**

Median age in months (range)	31 (11–>42)	24 (6–>42)	18 (2–38)	8 (6–28)	14 (6-7)	4.5 (3–6)	** Median** **15 mo.**	41 (18–>42)	25 (6–>42)	16 (3–>42)	14 (5–26)	14 (2–22)	9 (9)	**Median** **18 mo.**

Average lesion score in animals with culture-positive lesions (range)	3.7 (1–7)	3.1 (2–5)	2.4 (2–4)	2.1 (1–4)	2.2 (2-3)	2.5 (2.5)	**Average** 2.8^∗3^	3.6 (2–7)	3.2 (2–7)	3.0 (1–6)	2.8 (2–6)	3.7 (2–5)	3.1 (3.1)	**Average** 3.1^∗3^

^∗1^Pigs born in 2012 were not considered in subsequent cohort-delineated TB analyses due to too few animals.

^∗2^Overall totals exceed the sum of the individual males and females because pig gender was not recorded in all cases.

^∗3^Lesion scores were only recorded for culture-positive cases and only for those animals in which accurate lesion descriptions had been obtained; hence averages here were calculated from 102 out of a total of 221 Molesworth pigs and from 94 out of a total of 131 Clarence pigs for which lesions scores were recorded and for which the culture result was positive.

**Table 3:** Comparison of TB prevalence rates among released sentinel and resident wild pigs in the Molesworth and Clarence study areas, following the release and recovery of 51 *M. bovis*-naïve sentinel pigs into the areas between May 2009 and July 2010.

	Number of pigs (gender)	Average (range) duration of postweaning exposure in days	TB prevalence as percentage (95% CI)	Average lesion severity score among culture-positive cases (95% CI)	AAI of new infections over the exposure period
Molesworth					
Released sentinels	40 (22f, 18m)	259 (147–361)	20.0 (7–33)	2.6 (0.7–4.5)	0.28
Residents	59 (30f, 29m)	219 (122–397)	33.9 (21–46)	2.0 (1.7–2.3)	0.42
Clarence					
Released sentinels	11 (6f, 5m)	180 (104–298)	54.5 (19–90)	2.0 (1.1–2.9)	1.11
Residents	17 (9f, 8m)	260 (92–366)	70.6 (46–95)	2.7 (2.0–3.5)	1.36

Fifty-one purpose-reared sentinel pigs were released in the two areas between May 2009 and July 2010 and then recovered at various times between January 2010 and April 2011 for necropsy, by which time the animals had received 104–361 days of exposure to the endemic force of infection. One hundred and fifty-three resident pigs were identified as having been born in the area between March 2009 and May 2010 (and thus susceptible to infection, postweaning, between May 2009 and July 2010) and, from these, 76 animals were selected as having similar exposure duration to the sentinels before they were killed and necropsied (i.e., they had been exposed to the same force of infection in the same area and over the same time period as the released sentinels).

## Abbreviations

AAI: Apparent annual incidence (of* M. bovis* infection)
CCI: ChewCard index
CI: (95%) confidence interval
CKRI: Capture/kill-rate index
GPS: Global positioning system
KDE: Kernel density estimator
NPCA: National Pest Control Agencies
NVL: No visible (TB) lesions
OIE: Office International des Épizooties
PoF: Proof of Freedom (software utility for predicting TB eradication probability)
TB: Tuberculosis (due to* Mycobacterium bovis*)
TBFree NZ: TBFree New Zealand (management agency responsible for TB control and eradication in New Zealand)
TCI: Trap-catch index
VHF: Very high frequency
WTI: WaxTag index
1080: Sodium fluoroacetate.
