# Supplementary material for: Assessing the Effectiveness of Tuberculosis Management in Brushtail Possums (Trichosurus vulpecula), through Indirect Surveillance of Mycobacterium bovis Infection Using Released Sentinel Pigs
Source: Vet Med Int. 2014 Apr 2;2014:361634. doi: 10.1155/2014/361634 (PMC3996883; doi:10.1155/2014/361634)
Supplement: Supplementary file 1 — In pigs, TB lesions were classified on a scale of 2–7 based on ascending lesion severity of the largest recorded lesion in the head region lymphatic system. In the absence of gross lesions, a score of 1 represented an animal bearing M. bovis-infected tissues while a score of zero represented a non-lesioned/non-infected animal. [file 361634.f1.pdf]

Supporting supplementary information

Scoring system used to classify severity of infection in head lymph nodes of individual pigs

Score	Appearance of the largest identifiable head-region lymph node lesion
0	No visible lesions and culture negative submaxillary nodes, or (rarely), culture-negative lesioned tissue
1	No visible lesions, culture positive
2	Single or multiple small lesions <0.5 cm
3	Single or multiple lesions 0.6–1.0 cm
4	Single or multiple lesions 1.1–2.0 cm
5	Single or multiple lesions 2.1–3.0 cm
6	Single or multiple lesions 3.1–4.0 cm
7	Single or multiple lesions >4.0 cm
